# The efficacy and safety of deucravacitinib compared to methotrexate, in patients with vulvar lichen planus who have failed topical therapy with potent corticosteroids: a study protocol for a single-centre double-blinded randomised controlled trial

**DOI:** 10.1186/s13063-024-08022-y

**Published:** 2024-03-12

**Authors:** Marlene Wijaya, Gayle Fischer, Rebecca Bronwyn Saunderson

**Affiliations:** 1 Department of Dermatology, Royal North Shore Hospital, Reserve Rd, St Leonards, New South Wales Australia; 2 The University of Sydney, Northern Clinical School, St Leonards, New South Wales Australia

**Keywords:** Vulvar lichen planus, Deucravacitinib, Methotrexate, Randomised clinical trial

## Abstract

**Background:**

Vulvar lichen planus (VLP) is a chronic vulvar dermatosis that is difficult to treat and can severely impair quality of life in the absence of adequate treatment. There is a lack of high-quality evidence to direct therapy for VLP. This randomised controlled trial will be the first double-blinded study comparing systemic treatments in VLP and aims to investigate the safety and efficacy of deucravacitinib compared to methotrexate, in patients with VLP who have failed treatment with potent topical corticosteroids.

**Methods:**

A total of 116 women aged ≥ 18 years with moderate to severe VLP (Genital Erosive Lichen Planus (GELP) score ≥ 5) will be recruited. All participants will initially be treated with Diprosone® OV daily, and their outcome will be assessed using the GELP score. At 8 weeks’ follow-up, responders (GELP < 5) will be continued on Diprosone® OV. Non-responders (GELP ≥ 5) will be randomised 1:1 in a blinded fashion to receive (i) methotrexate 10 mg weekly + placebo tablet twice daily + folic acid 5 mg weekly or (ii) deucravacitinib 6 mg twice daily + placebo tablet weekly + folic acid 5 mg weekly. The primary endpoint is the difference in the mean change of GELP scores from baseline to week 32 between deucravacitinib and methotrexate groups.

**Discussion:**

High-quality evidence guiding the management of women with VLP is lacking. Once completed, this will be the first double-blinded RCT to compare systemic treatments in VLP. The results of this study will provide valuable, high-quality data to guide second-line therapy options for VLP that is recalcitrant to potent topical corticosteroids.

**Trial registration:**

Australian New Zealand Clinical Trials Registry ACTRN12623000682640. Registered on 26 June 2023.

## Administrative information

Note: the numbers in curly brackets in this protocol refer to the SPIRIT checklist item numbers. The order of the items has been modified to group similar items (see http://www.equator-network.org/reporting-guidelines/spirit-2013-statement-defining-standard-protocol-items-for-clinical-trials/).

**Table:** 

Title {1}	The Efficacy and Safety of Deucravacitinib to that of Methotrexate, in Patients with Vulvar Lichen Planus who have Failed Topical Therapy with Potent Corticosteroids: A Study Protocol for a Single Centre Double-Blinded Randomised Controlled Trial
Trial registration {2a and 2b}.	Australian New Zealand Clinical Trials Registry, ACTRN12623000682640. Registered 26 June 2023, https://www.anzctr.org.au/Trial/Registration/TrialReview.aspx?id=384798&isClinicalTrial=False
Protocol version {3}	Version 1.3, 26 January 2023
Funding {4}	Study funding and investigational product provision were awarded by Bristol-Myers Squibb.
Author details {5a}	Marlene Wijaya, BMed, MD, MPhil^1,2^; Gayle Fischer, MBBS, MD, FACD^1,2^, Rebecca Bronwyn Saunderson, BMedSci (Hon1), MBBS (Hon), MPhil (Cantab), FACD^1,2^ ^1^ Department of Dermatology, Royal North Shore Hospital, St Leonards, New South Wales, Australia ^2^ The University of Sydney, Northern Clinical School, St Leonards, New South Wales, Australia
Name and contact information for the trial sponsor {5b}	Dr Rebecca Bronwyn Saunderson P: + 61 2 9966 0625
Role of sponsor & funder {5c}	The sponsor oversees and maintains authority in all aspects of the trial, including the trial design, data collection, management, analysis and interpretation, writing of the report and the decision to submit the report for publication. The funder provides study funding and investigational product provision.

## Introduction

### Background and rationale {6a}

Since the 1900s, women’s health has been recognised as an important public health issue. Australia’s first National Women’s Health Policy was created in 1989, with one of the underpinning principles being “a lifespan approach without undue focus on the reproductive years” [1]. The listed priority areas included reproductive health and sexuality, mental and emotional health, and the health of ageing women. This reflected public awareness that women’s health requires a holistic approach and has changing needs throughout the stages of life.

Since then, considerable progress has been made in women’s health, such as access to contraception, access to regular cervical screening testing, mammography and in vitro fertilisation.

Importantly, the Australian National Women’s Health Strategy 2020–2030 reported it is paramount that we “identify and focus on the collection of more detailed and nuanced data, particularly for women and girls in underrepresented population groups and with less prevalent conditions, to inform health policy development and program delivery and to break the ‘cycle of invisibility’” [2].

Unfortunately, diseases affecting the genitals remain ‘invisible’ and an unmet need. This is despite genital diseases having one of the greatest influences on quality of life; genital diseases have substantial impacts on self-esteem, psychological well-being, physical activities, influence body image, clothing choices, disturb sleep, impair activities of daily living, leisure activities, the ability to work and relationships and sexual function [3–5].

Genital diseases are often overlooked in clinical practice, either due to reticence by patients to bring their embarrassing condition to attention, or clinicians, who feel ill-equipped to address them. Patients often present many years after suffering silently and, when they do, are often screened for sexually transmitted infections, repeatedly. Patients remain invisible and alone.

Vulvar lichen planus (VLP), the disease in focus in the presented study protocol, is a chronic inflammatory dermatosis characterised by erythema, erosions and hyperkeratosis on the vulva with possible vaginal involvement [6, 7]. It has a predilection for peri-/post-menopausal women in the fifth to sixth decades of life [8, 9]. The exact prevalence of VLP is unknown. It is estimated to be 3.7% from a study of 3350 women presenting to a vulvar clinic [8]. However, the actual prevalence in the general population is likely to be higher due to underdiagnosis.

Patients report significant vulvar pruritus, pain, burning sensation, discharge and dyspareunia, which leads to debilitating effects on sexual function and other aspects of life and reduces quality of life [4, 10, 11]. Ongoing disease activity can lead to vulvovaginal scarring and malignancy [10–12].

VLP is particularly difficult to treat. Unfortunately, despite the recognised disease sequelae and impact on quality of life, there are limited studies on direct therapy for VLP. The first-line treatment for VLP is potent topical corticosteroids, but approximately 20–40% of patients require second-line treatment with systemic immunosuppression to control their disease [9, 13]. Some of the systemic medications that have been used to treat VLP include prednisolone, methotrexate, mycophenolate mofetil, azathioprine, hydroxychloroquine, cyclosporine, rituximab, tildrakizumab, secukinumab, ustekinumab, adalimumab, tofacitinib and apremilast with varying results [14–26]. The best-documented agent appears to be methotrexate, with authors reporting a moderate efficacy and safety profile [15, 27–29]. However, these studies were either retrospective or small case series. There is no high-quality evidence, such as RCTs, to compare and guide second-line systemic treatments in VLP.

The pathogenesis of VLP is not completely understood, but there is evidence of involvement of tyrosine kinase 2 (TYK2)-mediated pathway. Cytokines, such as interleukin (IL)-12, IL-23 and type 1 interferon (IFN), signal through TYK-2, promoting the release of effector cytokines such as IL-17, which is known to play a role in the pathogenesis of various immune-mediated diseases [30]. IL-12/23 inhibitor ustekinumab and IL-23 inhibitor guselkumab have shown promise in the treatment of lichen planus, and tildrakizumab, an IL-23 inhibitor, was recently reported in the successful treatment of 24 women with VLP [19, 21]. Rapid clinical improvement and a marked decrease in IL-17A expression were demonstrated in a patient with VLP following a 12-week treatment with secukinumab, an IL-17A inhibitor [21].

One drug known to modulate the TYK-2 pathway is deucravacitinib (Sotyktu), a novel, first-in-class, oral, selective, allosteric TYK-2 inhibitor. It has been approved in Australia for adult patients with moderate-to-severe plaque psoriasis who are candidates for systemic therapy or phototherapy, and is being studied in psoriatic arthritis, alopecia areata and systemic lupus erythematosus with promising results [31–34].

### Objectives {7}

Given the lack of high-quality evidence to guide the use of second-line systemic treatments in VLP, the evidence of involvement of TYK2-mediated pathway in VLP and that the best documented systemic agent in VLP to date is methotrexate, the aim of this study is to conduct a double-blinded RCT comparing the efficacy and safety of deucravacitinib to that of methotrexate, in patients with VLP who have failed first-line treatment with potent topical corticosteroids. Once completed, this will be the first double-blinded RCT comparing systemic treatments in VLP. The results of this study will guide the management of patients with this chronic, long-neglected and particularly difficult-to-treat condition, addressing what is currently an unmet need.

### Trial design {8}

This study is a double-blinded, single-centre RCT with a parallel group design. The trial will take place over 36 weeks. Recruited participants will be prescribed potent topical corticosteroids with Diprosone® OV (betamethasone dipropionate 0.05% ointment in optimised vehicle) daily. At 8 weeks’ follow-up, those who have not responded to Diprosone® OV, defined by a Genital Erosive Lichen Planus (GELP) score [7] ≥ 5, will be randomised in a 1:1 ratio and blinded fashion to either the methotrexate or deucravacitinib group. The treatment will conclude at week 32. There will be a follow-up visit 30 days following the end of the treatment period.

## Methods: participants, interventions and outcomes

### Study setting {9}

This RCT will be undertaken at dermato-gynaecology clinic rooms of dermatologists who specialise in disorders of the vulva in Sydney, Australia.

### Eligibility criteria {10}

The inclusion criteria are females aged ≥ 18 with a diagnosis of moderate-to-severe VLP, demonstrated by a GELP score ≥ 5, of which erythema and pain must score ≥ 1. The diagnosis needs to be confirmed by either histopathology or fulfilling diagnostic criteria developed by Wu et al. [6] or Simpson et al. [35].

The exclusion criteria are:

- Those with a lichen sclerosus/lichen planus overlap.
- Patients receiving other systemic immune-modulating therapy within the previous 4 weeks.
- Has cancer or a history of cancer (solid organ, hematologic including myelodysplastic syndrome or melanoma in situ) or lymphoproliferative disease within the previous 5 years (other than resected cutaneous basal cell or squamous cell carcinoma, or carcinoma of the cervix in situ that has been treated with no evidence of recurrence).
- Premalignant cervical or vulvar disease.
- Live vaccine administration within the last 4 weeks.
- The concomitant use of strong CYP3A4 enzyme inducers.
- Inadequate birth control (if pre-menopausal), pregnancy, planning pregnancy during the study period and/or breast-feeding.
- A past medical history of depression and/or suicidal ideation. If these are reported on screening questions, then the subject will undergo a Patient Health Questionnaire 8 items (PHQ-8) excluding those when the total score is ≥ 15.
- Patients with severe renal/liver impairment or concurrent medications that would interact with the trial medications.
- Patients with active tuberculosis (TB), including any symptoms or signs, or imaging showing active TB, or latent TB determined by a positive IFN-gamma release assay and including current treatment for latent TB.
- Patients with other serious infections, defined by evidence of active infection or febrile illness within 7 days prior to day 1; a history of serious bacterial, fungal, or viral infection requiring hospitalization and/or intravenous antimicrobial intervention within 60 days prior to day 1; any ongoing evidence of chronic, bacterial infection (e.g. chronic pyelonephritis, chronic osteomyelitis, chronic bronchiectasis); a history of prosthetic joint infection where the prosthesis was not removed; active herpes simplex virus or herpes zoster infection at day 1; positive test for hepatitis B virus (positive HBsAg, HBcAb); evidence of, or test positive for, hepatitis C virus (HCV) at screening (anti-HCVAb), positive for human immunodeficiency virus by antibody testing (HIV-1 and HIV-2 Ab).
- Any history of known or suspected congenital or acquired immunodeficiency state or condition that would compromise the participant’s immune status (e.g. history of opportunistic infections [e.g. *Pneumocystis jirovecii* pneumonia, histoplasmosis, or coccidioidomycosis], history of splenectomy, primary immunodeficiency).
- Severe SARS-CoV-2 infection (e.g. worsened shortness of breath and pneumonia) within 4 weeks prior to screening. Additionally, in the case of prior SARS-CoV-2 infection, symptoms must have completely resolved, and based on investigator assessment in consultation with the clinical trial physician, there are no sequelae that would place the participant at a higher risk of receiving investigational intervention.
- Any history of hypersensitivity to the active substance(s) or to any of the excipients of deucravacitinib or methotrexate.
- Participation in another trial that could affect the current study.

### Who will take informed consent? {26a}

The recruiting investigators will obtain written informed consent from all eligible and interested participants prior to conducting any trial-related procedures. The investigators will ensure that participants are clearly and fully informed about the purpose, potential risks, alternative treatments available and other important matters regarding the clinical trial.

### Additional consent provisions for collection and use of participant data and biological specimens {26b}

There will be a separate consent form for tissue sampling in matched healthy controls for biomarker assessments.

## Interventions

### Explanation for the choice of comparators {6b}

Methotrexate was chosen as the comparator, as this is the best-documented agent for VLP [15, 27–29]. It is worth noting however that these studies were either retrospective or small case series, emphasising the scarcity of high-quality studies in VLP.

### Intervention description {11a}

All participants will initially be prescribed potent topical corticosteroids with Diprosone® OV daily. At 8 weeks’ assessment, responders (GELP < 5) will be continued on Diprosone® OV. Non-responders (GELP ≥ 5) will be randomised 1:1 in a blinded fashion to:

1. Oral methotrexate 10 mg weekly + oral placebo twice daily + oral folic acid 5 mg weekly, OR
2. Oral deucravacitinib 6 mg twice daily + oral placebo weekly + oral folic acid 5 mg weekly.

The treatments will be continued until week 32.

### Criteria for discontinuing or modifying allocated interventions {11b}

A participant may withdraw from the study for any reason at any time.

Permanent discontinuation from the study will take place if a participant:

- Requires oral corticosteroids for more than four consecutive weeks.
- Requires rescue treatment with a non-corticosteroid systemic agent (including but not limited to cyclosporine, methotrexate, mycophenolate mofetil, or azathioprine) or injectable or parenteral corticosteroid.
- Develops clinically significant abnormal laboratory results or adverse events (AEs), which rule out continuation of the study drug, as determined by the investigator.
- Becomes pregnant or plans to become pregnant during the study.
- Develops active TB during the study.
- Develops malignancy, except for localised non-melanoma skin cancers or carcinoma of the cervix in situ.
- Has a confirmed diagnosis of deep vein thrombosis, pulmonary embolus, or non-cardiac, non-neurologic arterial thrombosis.
- Takes prohibited medications when the continuation of the study drug would place the subject at risk, as determined by the investigator.
- Significantly non-compliant with study procedures, which would put the participant at risk for continued participation in the trial as determined by the investigator.

### Strategies to improve adherence to interventions {11c}

Participants will be provided with a study drug diary and asked to return drug containers to monitor adherence. The investigators will also remind participants of the treatment regimen at the end of every study visit.

### Relevant concomitant care permitted or prohibited during the trial {11d}

The following concomitant medications are permitted during the trial:

- Medications to treat chronic or acute conditions (with the exception of the prohibited medications listed below).
- Inhaled, ophthalmic drops and nasal corticosteroid formulations.
- Oral corticosteroids are permitted for rescue treatment, however may not exceed four consecutive weeks.
- Non-live vaccines.

The following concomitant medications or procedures are prohibited during the trial:

- Investigational product from another clinical trial.
- Concomitant systemic agents, including but not limited to methotrexate, cyclosporine, azathioprine, PDE-4 inhibitors, mycophenolate mofetil, biologic therapies and biosimilar versions of biologic drugs.
- Topical treatments for VLP other than the one prescribed in the study (Diprosone® OV).
- Intravenous, intramuscular and intralesional corticosteroids for VLP management.
- Live vaccines are prohibited during the study and up to 4 weeks following the last dose of the study drug.
- Medical and recreational cannabis.
- Traditional Chinese medicine.
- Strong CYP3A inhibitors or inducers.
- Elective surgery. If the subject must undergo emergency surgery, the study drug should be interrupted at the time of the surgery.

### Provisions for post-trial care {30}

Participants will return to standard clinic care post-trial.

### Outcomes {12}

The primary endpoint is the difference in mean change of GELP scores from baseline (week 8) to week 32 between deucravacitinib and methotrexate treatment groups.

The secondary endpoints will explore mean changes in:

- Vulvar Quality of Life Index (VQLI) [3] at weeks 8, 24 and 32
- Weekly use of topical corticosteroid, collected from the patient diary, and the number of 30-g tubes used
- General Health Questionnaire-28 (GHQ-28) [36] at weeks 8, 24 and 32
- Physician Global Assessment (PGA) [37] at weeks 8, 24 and 32
- Patient Global Assessment (PtGA) [37] at weeks 8, 24 and 32
- Immunological changes and expression of cytokines in serum (weeks 0, 8, 32) and tissue (selected participants) at weeks 0 and 32

### Participant timeline {13}

**Fig. 1:**
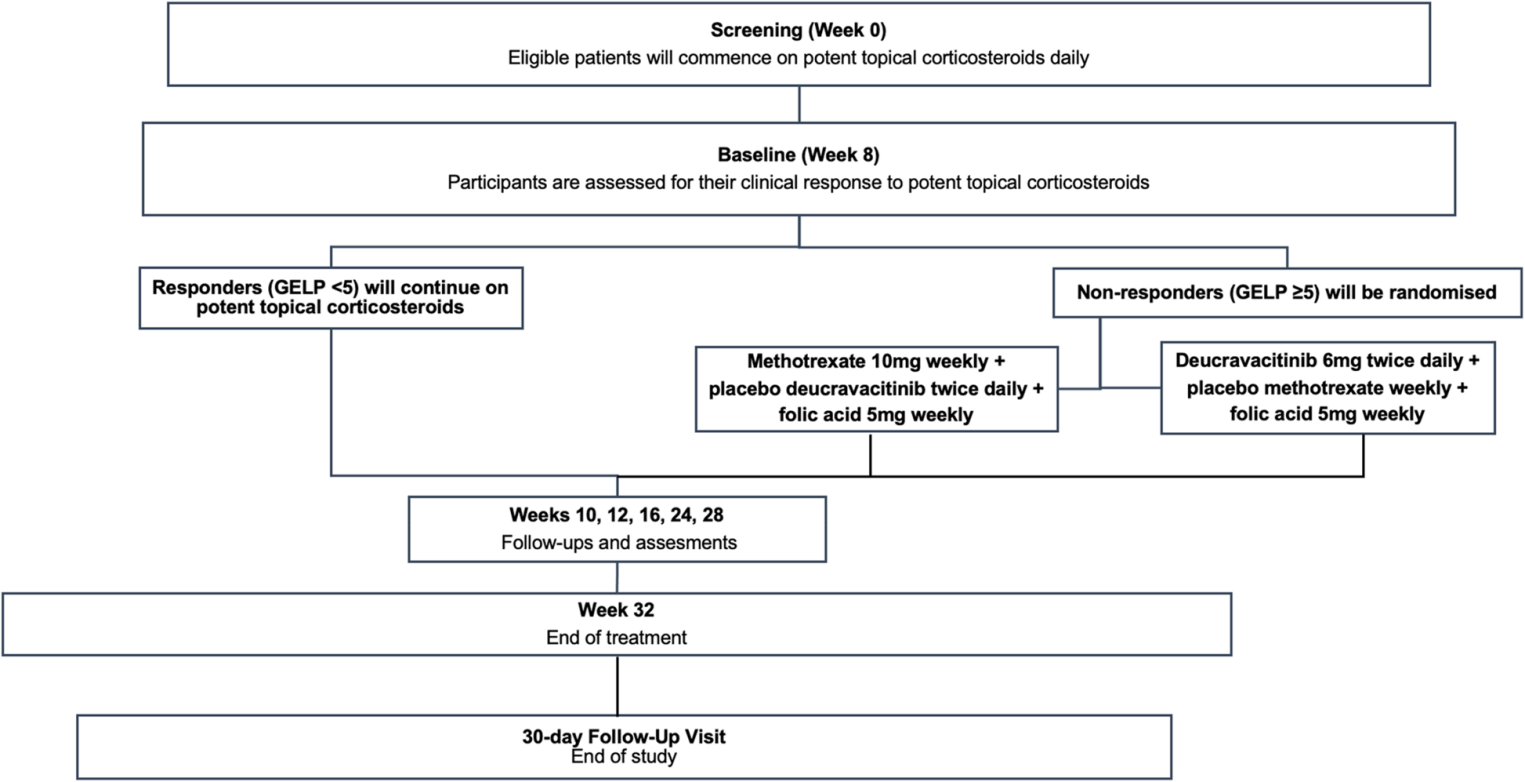
Participant timeline

The participant timeline is shown in Fig. 1. A follow-up visit will take place 30 days (± 1 week window) after the end of the treatment visit (week 32) or after a premature discontinuation visit.

**Fig. 2:**
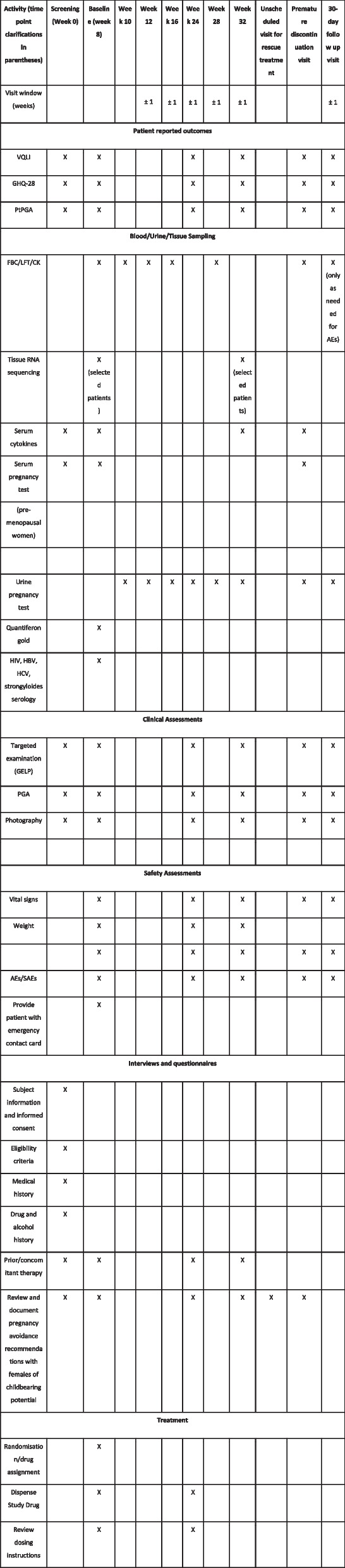
SPIRIT Figure outlining the schedule of activities for each study visit

The schedule of activities for each study visit is shown in Fig. 2 (SPIRIT Figure).

### Sample size {14}

A total of 116 patients will be recruited. The sample size was determined using a *T*-test for independent means (STATA, version 17.0) [38]. We based the calculation of our sample size on a previous RCT comparing the mean change of GELP scores between two intervention groups after 24 weeks of treatment [7]. As the mean reduction in GELP scores in their intervention group of interest was equivalent to a standardised mean difference (SMD) of > 0.80 (large effect size based on Cohen’s* d* measure [39]), we used an SMD of 0.70 (moderate to large effect size) in our calculation. The result of the analysis indicated that a total of 58 participants, or 29 in each deucravacitinib and methotrexate group, will be required to provide 80% statistical power with a two-sided 5% significance level to detect an SMD of 0.70 in the mean change of GELP scores from baseline (week 8) to week 32 between the two intervention arms.

According to the literature, approximately 20–40% of VLP patients would require second-line treatment with systemic immunosuppression to control their disease [9, 13]. In the study by Fahy et al. [9], over 40% of patients required systemic therapy. Based on these, we estimated that half of the participants will be non-responders, and therefore, a total of 116 participants will need to be recruited to provide a sample size of 58 for both the deucravacitinib and methotrexate groups or 29 in each treatment arm.

### Recruitment {15}

The study site is one of the very few dermato-gynaecology centres in New South Wales and receives a high volume of referrals for patients with vulval disorders, including VLP.

## Assignment of interventions: allocation

### Sequence generation {16a}

The methotrexate and deucravacitinib treatment groups will have a 1:1 ratio of allocation, generated using appropriate statistical software.

### Concealment mechanism {16b}

Treatment allocation will be performed by a separate party, who will feed this data to REDCap. The investigators will not have access to this data on REDCap. Hence, the allocation sequence will be concealed from the investigators and participants. Container labels will use KIT numbers.

### Implementation {16c}

A separate party, who is not involved in other aspects of the study, will generate the allocation sequence and create the randomisation list. The investigators will enrol participants and remain blinded to the treatment allocation.

## Assignment of interventions: blinding

### Who will be blinded {17a}

The investigators, outcome assessors, trial participants and statistician assisting with the data analysis will be blinded.

### Procedure for unblinding if needed {17b}

Unblinding of the treatment allocation will be allowed in events such as medical emergencies, where the safety and well-being of the participant are of concern. The pharmacy will be contacted to assist with unblinding.

## Data collection and management

### Plans for assessment and collection of outcomes {18a}

Study data, including clinical assessments, patient-reported outcomes and other study instruments, will be centralised and entered directly into an electronic data capture (EDC) platform, REDCap. All personnel collecting data will be trained. Investigators will review all data for accuracy and completion.

### Plans to promote participant retention and complete follow-up {18b}

Participants will be provided with emergency contact to assist with prompt management of issues and concerns raised by participants during the trial. If a participant does not attend their scheduled visit, every effort will be made to contact the participant and reschedule their study visit. A follow-up visit will take place 30 days (± 1 week window) after the end of the treatment visit (week 32), or after a premature discontinuation visit.

### Data management {19}

Data will be centralised and entered directly into REDCap, a secure platform supported by the Northern Sydney Local Health District Human Research Ethics Committee.

### Confidentiality {27}

Personal information about potential and enrolled participants will be entered into REDCap, a secure platform supported by the Northern Sydney Local Health District Human Research Ethics Committee.

### Plans for collection, laboratory evaluation and storage of biological specimens for genetic or molecular analysis in this trial/future use {33}

Serum sampling for biomarker evaluation will be collected from all VLP participants at weeks 0, 8 and 32 (or premature discontinuation visit). For tissue sampling, this will be performed on 10 participants with VLP (weeks 0 and 32) and 10 matched healthy controls (once only). Each tissue sampling will involve taking two 3-mm punch biopsies.

The planned investigations include:

(i) Serum cytokine mRNA analysis involved in TYK-2-mediated pathway, e.g. IFN-type I (IFN-a), IL-12, IL-23 and IL-17, and downstream pathways including CXCL9/10/11 and IFN-gamma
(ii) Tissue cytokine mRNA analysis involved in TYK-2-mediated pathway, e.g. IFN-type I (IFN-a), IL-12, IL-23 and IL-17, and downstream pathways including CXCL9/10/11 and IFN-gamma
(iii) Immunohistochemistry with T-cell markers on tissue

## Statistical methods

### Statistical methods for primary and secondary outcomes {20a}

The data will be presented as mean with standard deviation, median with interquartile range, or frequencies with percentages as appropriate. Analysis of covariance (ANCOVA) will be used to compare the mean change in GELP scores from baseline and week 32 between the deucravacitinib and methotrexate groups. Other endpoints will be analysed using independent samples *T*-test, Mann–Whitney *U* test, Fisher’s exact test, ANCOVA model or linear mixed effects model as appropriate. All statistical tests will be performed at a 0.05 level of significance.

### Interim analyses {21b}

N/a—there is no plan for interim analysis.

### Methods for additional analyses (e.g. subgroup analyses) {20b}

We will perform intention-to-treat (ITT) and per-protocol set (PPS) analyses. The number and proportion of screened, randomised, treated and analysed participants will be evaluated.

### Methods in analysis to handle protocol non-adherence and any statistical methods to handle missing data {20c}

The percentage of missing values for all the variables at each time point will be reported. For the primary outcome analysis, the missing values will be imputed using the multiple imputation method. Ten sets of imputed data will be created and analysed using the analysis method described for the primary outcome. The mean difference in mean change from baseline to week 32 between the methotrexate and deucravacitinib groups from the ten imputed analyses will be combined to obtain a pooled common mean difference and 95% CI. A complete case analysis will also be performed to assess the sensitivity of the magnitude and significance of the effects compared. All statistical tests will be performed at a 0.05 level of significance.

### Plans to give access to the full protocol, participant-level data and statistical code {31c}

These will not be available for public access.

## Oversight and monitoring

### Composition of the coordinating centre and trial steering committee {5d}

The coordinating centre is the Dermatology Research Department at Royal North Shore Hospital, Sydney, Australia. The team will be responsible for running the day-to-day operations of the trial and will meet at regular intervals to discuss the study progress.

### Composition of the data monitoring committee, its role and reporting structure {21a}

There will not be a separate data monitoring committee. However, an independent Medical Monitor will be appointed.

### Adverse event reporting and harms {22}

AEs and serious adverse events (SAEs) will be graded in severity with the Common Terminology Criteria for Adverse Events (CTCAE) [40]. The investigators will monitor each subject for clinical and laboratory evidence of AEs throughout the study duration. All AEs will be followed to a satisfactory conclusion.

### Frequency and plans for auditing trial conduct {23}

The study investigators will closely monitor the trial and meet at regular intervals to discuss the study progress and any issues arising during the conduct of the trial.

### Plans for communicating important protocol amendments to relevant parties (e.g. trial participants, ethical committees) {25}

Any protocol amendments will only be enacted following approvals from the study funder (Bristol-Myers Squibb) and the Northern Sydney Local Health District Human Research Ethics Committee.

### Dissemination plans {31a}

Study findings will be disseminated in peer-reviewed medical journals and local and/or international conferences.

## Discussion

High-quality evidence-directing therapy in VLP is lacking. Clinicians often base the choice of treatments for their VLP patients on clinical experience, anecdotal observations, case reports and small case series.

So far there have been very few RCTs exploring treatments for women with VLP. The RCT by Helgesen et al. compared vulvovaginal photodynamic therapy to topical corticosteroids [7]. Simpson et al. conducted the “hELP” RCT, investigating the utility of systemic therapy in addition to potent topical therapy [41]. The trial involved four arms: (1) prednisolone and clobetasol propionate 0.05%, (2) hydroxychloroquine and clobetasol propionate 0.05%, (3) methotrexate and clobetasol propionate 0.05% and (4) mycophenolate mofetil and clobetasol propionate 0.05%. However, the study was open-label and had to be stopped prematurely, as they did not reach the recruitment target of 40 participants in their initial 12-month pilot study [42]. Their commentary provided valuable insights into the potential challenges that investigators may face when running a clinical trial in patients with specific demographics and relatively rare conditions. Nevertheless, we are optimistic that these challenges can be overcome in our RCT, which we are conducting in a dermato-gynaecology centre with a large patient base and a high referral rate. Most recently, Skullerud et al. published the study protocol of their double-blinded RCT [20]. However, their study compared apremilast with placebo and not other systemic treatments. Hence, to our knowledge, this will be the first double-blinded RCT comparing systemic treatments in VLP patients.

The results of this RCT will provide clinicians with high-quality evidence to guide second-line systemic treatments in VLP and provide data on the safety and efficacy of deucravacitinib in the management of patients with this chronic and particularly difficult-to-treat condition.

## Trial status

The trial follows protocol v1.3 dated 26 January 2023.

Participant recruitment will begin in October 2023 and aimed to be completed in October 2025.

## Data Availability

The trial dataset will only be available to the study investigators and statistician. The dataset is kept in REDCap, and the access requires approval from the REDCap administrator. Only personnel associated with the study will be able to gain access to the project. Further, REDCap requires login details with a password and two-factor authentication to enter its platform.
